# Oral Health and Dental Care in Older Korean Immigrants: A Qualitative Study

**DOI:** 10.1093/geroni/igaa057.1079

**Published:** 2020-12-16

**Authors:** Yuri Jang, Min-Kyoung Rhee, Jeong Chung Hyeon, Eun Young Choi, Juyoung Park, Nan Sook Park

**Affiliations:** 1 University of Southern California, Los Angeles, California, United States; 2 Leonard Davis School of Gerontology, Los Angeles, California, United States; 3 University of South Florida, Tampa, Florida, United States

## Abstract

Many segments of the U.S. population continue to experience a disproportionate burden of oral disease and inequities in dental care, and older Asian immigrant populations are among those at high risk. Responding to the needs to attend to ethnic and geographic variations among older Asian Americans and to better understand contextual factors that shape their experiences of oral health and dental care, the present study conducted in-depth interviews with eighteen older Korean immigrants in the Los Angeles Greater area. The qualitative inquiries were theoretically guided by the three core categories of the Andersen’s (1968, 1997) health service model: oral health needs, service barriers, and service outcomes. Using the constant comparison method, themes and sub-themes within each category were derived. The eight themes emerged from the qualitative data were: (1) oral health problems, (2) perceived need, (3) insurance and finance, (4) language barriers, (5) social support, (6) knowledge and belief, (7) satisfaction with service, and (8) areas of improvement. The findings demonstrated varied experiences associated with oral health and dental care of older Korean immigrants and informed the development of services and programs responsive to the identified needs and barriers.

